# How German and Italian Laypeople Reason about Distributive Shortages during COVID-19

**DOI:** 10.3390/ijerph191912067

**Published:** 2022-09-23

**Authors:** Ronja Demel, Francesco Grassi, Yasaman Rafiee, Michael R. Waldmann, Annekathrin Schacht

**Affiliations:** 1 Institute of Psychology, Georg-August University, 37073 Goettingen, Germany; 2 Institute of Psychology, Humboldt University of Berlin, 10117 Berlin, Germany; 3 Leibniz Science Campus “Primate Cognition”, 37077 Goettingen, Germany

**Keywords:** moral reasoning, moral dilemmas, COVID-19, coronavirus, corona pandemic, triage, choice deferral

## Abstract

(1) Background: The COVID-19 pandemic provided a unique opportunity to investigate how moral reasoning is influenced by individuals’ exposure to a crisis and by personal, societal and temporal proximity. We examined how Italians and Germans judged different behaviors that arose because of the pandemic, which affected health and societal matters. (2) Methods: Over the course of four months and three assessment periods, we used an observational online survey to assess participants’ judgments regarding seven scenarios that addressed distributive shortages during the pandemic. (3) Results: Overall, there was no clear answering pattern across all scenarios. For a variation of triage and pandemic restrictions, most participants selected a mean value, which can be interpreted as deferring the choice. For the other scenarios, most participants used the extremes of the scale, thereby reflecting a clear opinion of the public regarding the moral issue. In addition, moral reasoning varied across the two countries, assessment periods, fear, and age. (4) Conclusions: By using scenarios that were taken from real-life experiences, the current study addresses criticism that moral research mostly relies on unrealistic scenarios that lack in external validity, plausibility, and proximity to everyday situations. In addition, it shows how lay people regard measures of public health and societal decision-making.

## 1. Introduction

The COVID-19 pandemic has affected the entire world and put everyone in a hitherto unfamiliar situation that has lasted longer than anyone initially imagined. The pandemic has led to millions of illnesses and deaths worldwide [1]; cf. https://covid19.who.int/ (accessed on 28 July 2022) and economic crises in the majority of countries [2]. Because of the pandemic, a multitude of distributive decisions arose that directly affected a large number of people. The current study aimed at understanding how people reason about moral dilemmas that have occurred in manifold situations during the COVID-19 pandemic. Understanding people’s behavior and willingness to adapt their behavior is necessary to develop future health policies and has societal and political implications for a state of emergency. Although a number of studies have investigated how people make decisions in small-scale virtual real-life dilemmas, e.g., [3,4], the COVID-19 pandemic provides a unique opportunity to study changes of moral intuitions about real-world dilemmas in a longitudinal design.

COVID-19 was first identified in December 2019 in China and spread to the whole world within weeks, causing the World Health Organization (WHO) to declare the outbreak as a pandemic in March 2020 [5]. The virus is characterized by an easy human-to-human transmission by air and is spread by symptomatic and asymptomatic infected people [6]. While most infections are mild with flu-like symptoms, there are more severe courses, especially for risk groups, which require intensive medical treatment. Given the lack of vaccines in the first year of the pandemic, recommended measures included various social distancing measures, lockdowns, and the wearing of masks. The effectiveness of these measures depends largely on the cooperation of the population and resulted in a number of far-reaching (moral) decisions on different levels regarding the public’s understanding for policy development and implementation. How the outbreak and spread of the COVID-19 pandemic have been handled has varied between countries. Italy is a European country that was hit particularly hard by the crisis very early on [7]. In an attempt to understand the bad progression of Italy compared to other European countries, two main explanations were put forward: for one, Italy has the most elderly population in Europe, with many people showing relevant concomitant diseases. In addition, Italy’s ICU/health care system quickly reached its capacity [8]. Consequently, a nationwide compulsory quarantine was implemented by the government between 9 March and 18 May 2020. Germany showed a different progression and severity during the first wave in March 2020. In comparison to other countries, but especially Italy, Germany had a lower case fatality rate with a significantly lower mortality rate [1]. During the first wave, the capacities of the health system were not exhausted. A nationwide lockdown to stop the spread of the disease was implemented in Germany on 22 March 2020 and lasted until 6 May 2020. As it has been shown, Germany and Italy have had a very different progression at the beginning of the pandemic, albeit being similar in various aspects (e.g., western European democracies, similar in size and population density, and high average age of population). However, what differs between the two countries is the capacity of hospital beds and emergency care. While Germany is above the European average, Italy is below it [9]. Therefore, the two countries are ideal for a cross-cultural comparison regarding shortages in medical and socio-economic issues.

The COVID-19 pandemic has raised several questions of ethical relevance, originating from its huge impact on the health of people, on the economy of societies and on the personal freedom of their citizens. So far, most of the psychological research on moral dilemmas has focused on fairly simple dilemmas (see [10], for an overview). A classic paradigm is the trolley dilemma, in which a runaway train that is heading toward a larger number of victims who would be killed, can be re-directed onto a different track where a smaller number of victims would lose their lives. A major criticism of trolley dilemmas is that they are very abstract and extreme in their nature. It is argued that they lack ecological validity, are sometimes taken rather humorously than seriously, and, most importantly, that they do not elicit the same psychological processes as real situations [11,12]. In contrast, the COVID-19 pandemic has affected people around the world in their real life. Using moral dilemmas that are directly related to the pandemic will therefore increase ecological validity and address shortcomings of previous studies.

In the past decades, numerous relevant factors have been identified that have been postulated as morally relevant in philosophical ethics [13]. All theories agree that alternative outcomes affect intuitions, which is in line with the predictions of both consequentialist and non-consequentialist theories e.g., [14,15,16,17]. However, besides outcomes, non-consequentialist factors including the type of action, distance, rights, and obligations in the society also proved important [10,16].

The present study takes into account such factors and additionally considers influences by emotional factors, demonstrated to play a particular role in a global crisis, on moral judgments. One of the central emotions that has been identified during the COVID-19 pandemic is fear, which arises when perceiving the threat of harm [18]. Several studies reported an increase in distress, worry, depression, and stress levels in the course of the COVID-19 pandemic [19,20,21,22,23,24,25]. Importantly, an increase of fear has also led to certain behavioral adaptations [22,23,26], for instance, more responsible and protective behavior [22,27,28]. However, Sobkow et al. [23] showed that worry can lead to divergent behaviors: while worry about health led to the higher adoption of hygiene and social distancing measures, people who worried about personal restrictions adopted fewer prevention measures. For a precise assessment, it is therefore important to specify what fear refers to.

Very early in the pandemic, it became clear that some people are at particular risk for a severe course of a COVID-19 infection. High-risk groups include people of higher age (>70 years) [29] and people suffering from several medical preconditions, such as hypertension, diabetes, cardiovascular disease, chronic respiratory disease, and cancer [30]. Although older people are at higher risk, they assessed the risk of infection as being smaller than younger people in a German [31] and in an Italian sample [32]. However, older people were also more willing to comply with preventive measures in both countries [33,34]. A bias towards unrealistic optimism about the pandemic was observed in several relatively young samples of participants [35,36], and might explain the discrepancy between official warnings and protective behavior in accordance with these warnings [37]. Other studies found age differences with regard to worry, with higher scores for younger people [19,27,38]. Taken together, these results indicate age to be a relevant predictor in how people assess and act in accordance with the suggested and implemented prevention measures.

One of the moral concepts with massively increased both public and academic attention in the COVID-19 pandemic is *triage,* which refers to the general distribution of scarce resources in health contexts. During the current pandemic, triage became best known for determining whether patients should be prioritized in intensive care [39]. Italy, among other countries, had to apply triage early on when hospitals reached capacity limits for ICU care [40]. Triage represents a challenging problem with severe moral implications, and has been argued to be a prominent example for the application of a utilitarian principle [17] because it aims to maximize the good (i.e., ensure survival for as many as possible). According to many triage protocols, limited resources should be prioritized to those who benefit most from the treatment rather than to those requiring it the most, e.g., the sickest patients [41]. Although triage had to be applied in the past, the corona pandemic uniquely poses new challenges to existing triage systems, and may need to be extended to several other medical contexts, such as in the prioritization of vaccinations.

In addition to prioritizing medical treatments, another morally charged question concerns the extent to which it is acceptable to emphasize one’s own protection over the common good. As outlined above, age is a risk factor for a severe course of a COVID-19 infection. At the same time, older medical professionals have still been required to work, and in the case of Italy they were even asked to return from retirement to help with the high number of infected people [42]. A recent study indicated that people have a moral responsibility to contribute to the mitigation of a crisis when they are able to do so [43]. However, even in hypothetical dilemmas, self-sacrifice is rare, e.g., [44,45]. Whether it is justifiable to ask elderly medical personnel to put themselves at risk to save others is again a question for which there seems no clear solution. It is a balancing act between the Hippocratic Oath that physicians take, on one hand, and self-serving preservation on the other.

As outlined above in the context of triage, there is a necessity to decide how to distribute common resources when societies are confronted with commodity scarcity [46]. Stockpiling has been reported to lead to supply disruptions during major disasters all over the world e.g., [47,48,49]. Due to the rapid spread of the COVID-19 pandemic in March 2020, people started to stockpile goods of daily need, arguably to be equipped for potential shortages and lockdown measures. The spike in demand led to immediate shortages of food and hygiene products [50]. For medical resources, shortages due to stockpiling (e.g., of face masks) was so severe that even medical facilities such as hospitals no longer had enough supplies to equip health care workers [51]. This shortage had massive implications, as it possibly led to higher infection rates among health care workers in Italy [42]. Nevertheless, in the current pandemic, stockpiling has been argued to reflect the psychological need to maintain (or restore) control [52], and might be understood as a form of self-preservation, particularly when potentially facing self-isolation [53]. Dysfunctional stockpiling, however, has egocentric and competitive facets [53] and honest humility, a measure for active cooperativeness, has been demonstrated to be negatively related to stockpiling [54]. While stockpiling is an example of commodity scarcity on the level of individual behaviors, the limited availability of common goods also raises the risk of economic speculation by producers and suppliers. Due to the increased demand for some products (e.g., hand sanitizers or facemasks) but also difficulties in the production or distribution during the COVID-19 crises, prices for high demand items increased rapidly and many companies adjusted their market strategies accordingly [55]. This raised the question of whether government authorities should be involved in protecting consumers and take action to hinder exploitive pricing practices.

Secondary effects, beyond the health implications, have become increasingly relevant in the public and political debate the longer the pandemic lasted. Numerous core areas of society have been affected, including the education sector and the economy [56]. Part of the moral implication of the COVID-19 crisis is that there are different types of losses that have to be traded off [57]. Weighing the extent of preventive measures (e.g., quarantines of contacts of infected persons) against the threat of safeguarding basic services and the impact on local and global economies has not eased with the pandemic development. While compliance with preventive measures was in general high at the beginning of the pandemic, it was reduced particularly in younger people who are less likely to be severely endangered by an infection [33,58]. In addition, there is evidence that people tend to grow tired of the adaptation of protective behavior, especially when it leads to economic losses for themselves [59]. How economic losses should be valued in relation to public health protection in the containment of COVID-19 is a moral issue of increasing societal relevance.

This study aims to gain a better understanding of how individuals would make morally intricate decisions directly related to their personal lives and societal issues during the COVID-19 pandemic. We wanted to understand how the dissimilarities in the outbreak in Germany and Italy would lead to different moral evaluations of distributive shortages and how they progress over time. Moreover, our aim was to investigate how pandemic-relevant socio-demographic factors, namely age and fear, would affect the moral evaluations. Understanding how these factors influence the public’s moral reasoning during a global health crisis is important for identifying how to gain support for political measures and how to manage the crisis in the best interest of the public. This is not only important for the ongoing pandemic but also for being better prepared for future crises.

With regard to the aims of our study, laypersons were asked to give their spontaneous judgments on vignettes describing pandemic-related moral dilemmas. Through a longitudinal design, we were able to measure changes in moral reasoning in parallel to the development of the pandemic over three assessment periods in Germany and Italy. In line with our pre-registered study design (osf.io/8ubcv, accessed on 14 March 2022), we were also collecting data in Iran but had to cancel data acquisition during the first assessment period due to unexpected problems.

We expected longitudinal changes in moral reasoning and differences between the two countries due to varying severity of the pandemic. During our first assessment period (March 2020), the situation was rather new in Europe and had only recently been declared a pandemic. Both Italy and Germany were already in or right before a first nationwide lockdown. Whereas in Germany the situation was still under control, the situation in Italy was already severe. At assessment periods two and three (two and four months later), the situation was more relaxed in both countries. Beyond the general differences between both countries and the assessment periods, we expected variance in the moral decisions based on the subjective fear and age of the participants. In an explorative manner, we examined subjective evaluations of the situation, self-reported changes of behavior, confidence in the situation over time, and subjects’ predictions of long-term consequences. The results and discussion of these measures can be found in the Supplementary Material.

## 2. Materials and Methods

The study is longitudinal and cross-cultural with three assessment periods including participants from Germany and Italy. The project was approved by the ethics committee of the Institute of Psychology of the University of Goettingen. The aims, design, and hypotheses were preregistered on the open science framework before data collection (https://osf.io/8ubcv/, accessed on 14 March 2022).

### 2.1. Participants

Participants were recruited online two days after the WHO declared a pandemic on 11 March 2020, due to the global spread of COVID-19. Data collection was online via the open-source framework *formr* (version 0.7.4) [60] in three assessment periods: T1 took place for 10 days from 13 March to 23 March 2020; T2 and T3 were 60 and 120 days after T1, respectively, with the same duration as T1. For each participant, the period to complete each questionnaire was 10 days.

For T1, 1713 adults (1053 Germans, 600 Italians, 60 others, *M_age_* = 31.80 (*SD* = 11.48) years, 29.54% male, 69.94% female, 0.52% other) responded to the online questionnaire that was posted on Facebook, Twitter and circulated via e-mail using the lab internal mailing list and participant databases in German and Italian (“Other” nationalities include participants where nationality and residence were not the same or participants whose nationality and residence did not match the target countries (Germany, Italy, and Iran). Data that was collected from Iranian participants was excluded completely because of unforeseen problems in data acquisition during T2 and T3 and is not considered here.). The same participants were invited to take part in the data collection for T2 and T3. A total of 1157 participants (727 Germans, 396 Italians, 34 Others, *M_age_* = 31.80 (*SD* = 11.68) years, 28.87% male, 70.87% female, 0.26% other) finished the questionnaire for T2, and a total of 889 participants (567 Germans, 301 Italians, 21 Others, *M_age_* = 32.19 (*SD* = 11.94) years, 27.56% male, 72.10% female, 0.34% other) finished T3 and therefore the entire study. The whole dropout rate was 48.10%.

### 2.2. Testing Procedure

Before data collection at each assessment period started, participants were asked to give their informed consent in accordance with the Declaration of Helsinki [61] by pressing an “I agree” button located beneath an explanatory letter. After the completion of the questionnaire, participants were fully informed about the aim of the study.

In assessment period one, participants provided their demographic information including age, gender, educational background, occupation, nationality, country of residence, and religiousness. In addition, participants were asked about their pre-existing health conditions, and whether they were infected or knew someone who was infected with COVID-19. All these items were presented at once on one page in a fixed order. Detailed information on demographics can be found in the supplementary material (Table S2). To invite participants to the subsequent test sessions, participants provided their email addresses, which were exclusively used for this aim.

Subsequently, participants answered three questions regarding their subjective experience of the COVID-19 pandemic, including “fear of being infected”, “fear of death for self or closely related persons”, and perceived confidence (please see Table 1). Answers were given on a rating scale, ranging from 0 to 100, by moving a slider, initially located in the middle of the scale. Lastly, participants were asked to morally evaluate alternative courses of actions in five moral scenarios that referred to the current pandemic. The scenarios were adapted from classical moral dilemmas and varied in how much they referred to personal or societal conflicts. They included items requiring a prioritization of patients in a triage setting (i), judgments on the acceptability of individual stockpiling (ii) and of requiring retired physicians to provide medical care (iii). There were two more scenarios asking for decisions on price gouging for specifically demanded products (iv), and on the need of market regulations (v). Please see Table 1 for details on the scenarios and scale labeling. The scenarios varied in style: some of them were adapted from two-alternative choice questions, others asked for the acceptability of an action. We used answer scales that were labelled differently, but all required answers on a continuum to assess the nuanced decisions and to make the scenarios comparable. Each moral scenario was presented on top of separate pages along with a slider on a scale from 0 to 100 below, with the slider initially located in the middle of the screen. Once subjects considered their answer final, they could click a “continue” button to move to the next item on a new page.

In assessment periods 2 and 3, we added two moral questions about the assessment of the scope (vi) and the strictness (vii) of the prevention measures and several additional questions regarding the situation assessment, behavior changes, the expected long-term consequences, and a refined fear assessment in accordance with the *Fear of Coronavirus-19* scale [62]. The description and results of the additional questions can be found in the supplementary material.

### 2.3. Statistical Analyses

Statistical analyses were performed using R statistical software (version 3.6.2, R Core Team, Vienna, Austria) and RStudio (version 1.2.5033, RStudio, Boston, MA, USA). Standard *p*-values of 0.05 were used as a cut-off for two-tailed distributions. Data were excluded when participants did not finish all three assessments (T1–T3) or the nationality or country of residence did not match the targeted countries. This resulted in a final sample of *N* = 820 participants that was used for the subsequent analyses (551 Germans, 269 Italians, *M_age_* = 32.46 (*SD* = 12.27) years, 27.68% male, 71.95% female, 0.37% other). COVID-19 related fear was calculated as the average of the participant’s rating of “fear of being infected” and “fear of death for self or closely related persons” for each assessment period.

A Generalized Linear Mixed Model (GLMM) with beta error structure and logit link function was used to model the response to each of the five moral scenarios presented at all assessment periods, as well as the response to the additional moral questions presented at T2 and T3 only. The models included country (Germany, Italy), assessment period (T1, T2, T3), and their interaction as fixed effects. The covariates age and fear and their interaction with country and assessment period were also included as fixed effects. Participant was included as a random intercept effect. Models regarding the moral scenarios presented at all assessment periods, i.e., T1–T3 also included random slope of fear within individual identity. Age was log-transformed, and subsequently age and fear were both z-transformed to a mean of zero and a standard deviation of one. The responses to all scenarios were scaled in the open interval 0–1.

To test the overall effect of the fixed effects country, assessment period, and their interaction, each full model was compared with a reduced model lacking these two fixed effects. The likelihood ratio of the full model was compared with the likelihood ratio test of the reduced model [63]. Collinearity among predictors was tested by determining the Variance Inflation Factor (VIF) in a standard linear model. Individual fixed effects of interactions were tested using likelihood ratio tests [64]; R function drop1, with argument ‘test’ set to ‘Chisq’. For all models, the main effect of predictors not involved in any significant interaction was tested by fitting a reduced model lacking those interactions.

## 3. Results

**Figure 1:**
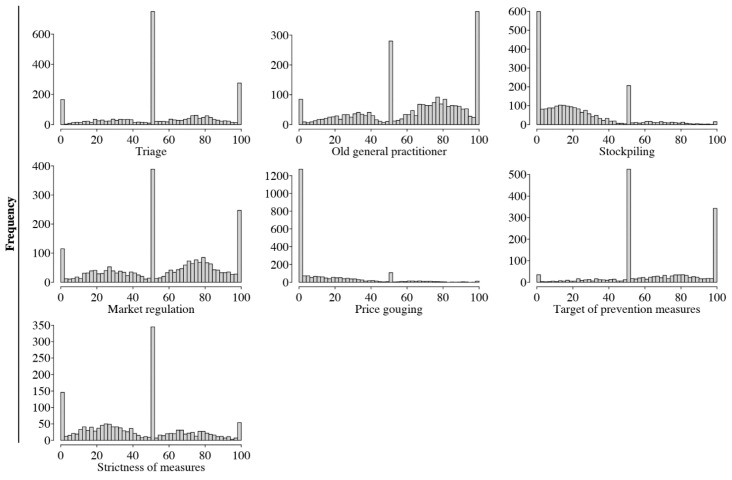
Absolute frequency of the responses to each moral scenario.

Distributions of the evaluative responses to each moral scenario are shown in Figure 1. For almost all scenarios, the distribution of the responses was strongly concentrated towards the center of the scale, with occasionally additional response peaks at one or the other extreme of the scale. The scenario regarding price gouging showed instead a higher density of responses towards the lower end of the scale, while the opposite pattern was observed for the scenarios regarding the old general practitioner.

Regarding the GLMMs used to model the response to the moral scenarios, a maximum VIF of 1.06 indicated no collinearity issues [65]. There was also no overdispersion issue in the observed data compared to any of the GLMMs. Summaries of the GLMMs can be found in the supplementary material (Table S3 for the full model of the seven moral scenarios; Table S4 for the respective reduced models).

### 3.1. Triage

**Figure 2:**
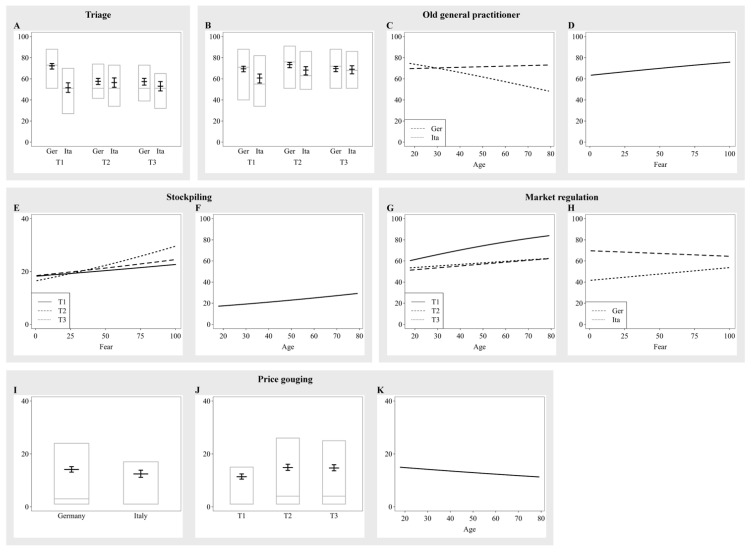
Estimates of the significant main effects and interactions for the ratings of the moral scenarios that were presented in all assessment periods. (**A**) Triage; (**B**–**D**) Old General Practitioner Dilemma; (**E**,**F**) Stockpiling; (**G**,**H**) Market regulation; (**I**–**K**) Price gouging.

Participants showed no clear tendency for older or younger patients when they were asked who to prioritize in scarcity of medical treatments (*M* = 55.84, *SD* = 27.81). During T1, Germans showed a stronger preference towards prioritizing older patients compared to Italians. However, this preference towards prioritizing older people was reduced for Germans during T2 and T3 to values similar to Italians who showed a less variable preference across assessment periods (*χ^2^* = 51.10, *df* = 2, *p* < 0.001; Figure 2A).

### 3.2. Old General Practitioner Dilemma

Participants were asked how acceptable they would find it if an old general practitioner would close his practice as he is at high risk of a severe course of a COVID-19 infection due to his age although there was a high demand of medical care due to the pandemic. Overall, participants found it acceptable, with ratings above 50 (*M* = 64.59, SD = 28.36). Italians found it less acceptable than Germans to close the practice at T1, but equally acceptable at T2 and T3, which was indicated by a significant interaction of country and assessment period (*χ^2^* = 8.84, *df* = 2, *p* = 0.012; Figure 2B). Furthermore, a significant interaction of country and age (*χ^2^* = 10.62, *df* = 1, *p* = 0.001) indicated that older Italians found it less acceptable to close the practice, whereas age did not play a role for Germans (Figure 2C). Moreover, a main effect of fear (*β* = 0.15, *SE* = 0.04, *p* < 0.001) indicated that participants who scored higher in fear found it more acceptable to close the practice (Figure 2D).

### 3.3. Stockpiling

For the stockpiling scenario, participants indicated how acceptable they would find it to stockpile for themselves even though this might lead to shortages for other people. Participants found it not acceptable to stockpile with a mean rating of *M* = 22.20 (*SD* = 22.06). A significant interaction of fear and assessment period (*χ^2^* = 6.52, *df* = 2, *p* = 0.038) indicated that higher fear led to a higher acceptance in stockpiling, which was even most prevalent in T2 and T3 (Figure 2E). Furthermore, older people found it more acceptable to stockpile (*β* = 0.16, *SE* = 0.03, *p* < 0.001; Figure 2F), independent of country and assessment period.

### 3.4. Market Regulation

There was no clear preference on whether the market should be regulated for scare medical supplies (*M* = 57.99, SD = 28.66). However, participants found it more acceptable to regulate the market with increasing age, especially in T1 compared to T2 and T3, which was indicated by a significant interaction of age and assessment period (*χ^2^* = 14.42, *df* = 1, *p* < 0.001; Figure 2G). In addition, fear led to a higher acceptance of market regulation in Italians, but not in Germans, for whom acceptance was lower when fear was higher (*χ^2^* = 6.45, *df* = 1, *p* = 0.011; Figure 2H).

### 3.5. Price Gouging

Regarding price gouging, participants indicated that they find it unacceptable if a company increases their profit margin during the pandemic (*M* = 14.54, *SD* = 20.73), with slightly higher acceptability scores of Italians compared to Germans (*β* = −0.15, *SE* = 0.08, *p* = 0.051, Figure 2I). In both countries, participants found price gouging less acceptable in T1 than in T2 and T3 (*β_T2_* = 0.31, *SE_T2_* = 0.05, *p* < 0.001; *β_T3_* = 0.29, *SE_T3_* = 0.05, *p_T3_* < 0.001; Figure 2J). In addition, older participants found it slightly less acceptable than younger participants (*β* = −0.07, *SE* = 0.04, *p* = 0.037; Figure 2K).

### 3.6. Target of Prevention Measures

**Figure 3:**
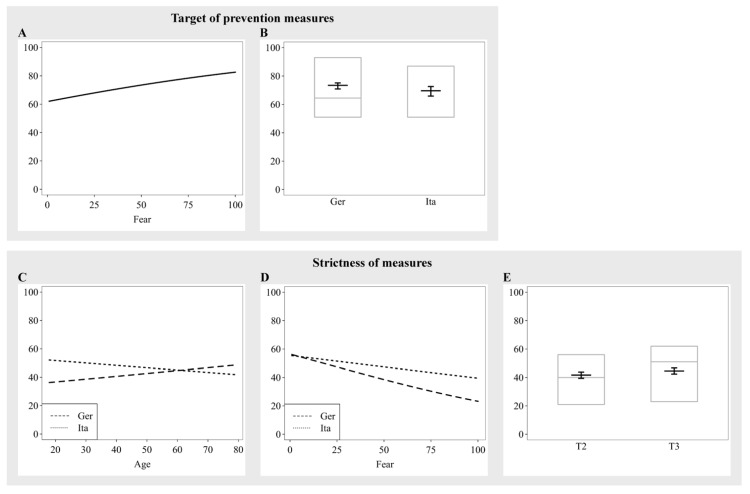
Estimates of the significant main effects and interactions for the ratings of the moral scenarios that were presented in assessment periods 2 and 3. (**A**,**B**) Target of prevention measures; (**C**–**E**) Strictness of measures.

When participants were asked whether prevention measures should be targeted to high-risk groups or the whole population, they showed a clear preference for applying it to everyone (*M* = 65.84, *SD* = 25.52). A significant main effect of fear (*β* = 0.28, *SE* = 0.04, *p* < 0.001) indicated that, in both countries and all assessment periods, fearful participants had a stronger preference toward protective measures for the entire population (Figure 3A). Moreover, we observed a weak tendency for Italians to prefer more specific restrictions to high-risk populations compared to Germans (*β* = −0.19, *SE* = 0.10, *p* = 0.051; Figure 3B).

### 3.7. Strictness of Measures

Participants showed no clear preference on how strict the protective measures (e.g., lockdowns) should be (*M* = 42.66, *SD =* 26.67). Younger Italians showed a preference for a relaxation of measures, while older Italians favored stricter measures to protect high-risk groups and reduce infections. There was an opposite pattern for Germans, with younger people favoring stricter lockdown measures compared to older people, indicated by an interaction of country and age (*χ^2^* = 6.61, *df* = 1, *p* = 0.010; Figure 3C). In addition, the interaction of country and fear (*χ^2^* = 8.58, *df* = 1, *p* = 0.003; Figure 3D) suggests that, while for participants from both countries, increasing levels of fear led to a stronger preference for stricter containment measures, this effect was stronger in Germans compared to Italians. Moreover, the main effect of assessment period was significant, indicating a stronger preference for stricter measures at T2 compared to T3, independently of country (*β* = 0.12, *SE* = 0.05, *p* = 0.015; Figure 3E).

## 4. Discussion

Our study aimed to investigate moral reasoning during a worldwide pandemic in a longitudinal and cross-cultural design. The COVID-19 pandemic has resulted in several resource shortages that dynamically changed over time and continuously led to decision conflicts. In our study, we focused on seven moral questions that arose due to these shortages and that were extensively discussed in the public and by experts. Across all moral scenarios, participants mostly selected values of 0, 50, and 100, with 50 being the most selected value. While values of 0 and 100 indicate strong preferences, values of 50 indicate that participants might have been unsure, too uninformed to make a choice, or that they thought of the question as too difficult for them to answer. Such choice avoidance or deferral has been reported in studies for trade-off generating questions where no single choice has a decisive advantage [66,67]. In our data, this pattern can mostly be observed for the scenarios concerning triage, target of prevention measures, and strictness of prevention measures. These problems have in common that they are not only trade-off questions that are difficult to make but that they also require additional knowledge and professional expertise. In real life, decisions about these issues are not made by lay people but by medical professionals and policy makers and have to follow guidelines to prevent unjust treatment. For triage, training about what critical care can do for realistic and informed decision-making is necessary [68]. For the target and strictness of prevention measures, both in Germany and Italy, there are clear regulations that forbid stricter measures for risk-groups based on, for example, age or precondition because they would be discriminatory (see Art. 2 Abs. 1 GG and Art 3 Cost). The fact that in our convenience sample these choices were mostly not made emphasizes the importance of having these guidelines and procedures by professionals available. Nevertheless, it is important to consider the public opinion in making these guidelines to build public trust and provide broader acceptance of the implemented policies [69]. Especially in a pandemic with many uncertainties, these decisions must be well communicated to the general public to ensure compliance with the proposed measures.

Although there was a general tendency in the triage scenario to not favor older or younger people, Germans had a slight preference to give the treatment to older people in T1 compared to Italians where more people indicated to give the treatment to younger people. This can likely be attributed to the differences in the severity of the outbreak during T1, where in Italy the hospitals were overloaded and triage had to be applied. In addition, in Italy, an age threshold was actually applied during the COVID-19 pandemic for resource allocation at the peak of the first wave [70]. Another study on the COVID-19 pandemic has found that people favored younger people in triage scenarios [71]. In moral judgments, it has been found that people make more lenient judgments when they gained from the behavior [72]. In addition, individuals’ opinion on fair resource allocation is strongly influenced by their self-interest [73]. Such a self-serving bias might have also driven the lower ratings for Italians in T1 as the more severe outbreak at the time in Italy compared to Germany made it more likely for (younger) Italians to need access to intensive medical care.

While triage is a moral example in which healthcare professionals are confronted with decisions on other people, they have also been faced with moral decisions potentially affecting their own well-being. Medical professionals are often willing to sacrifice their own comfort for their patients. However, they should not be placed at unjustifiable high personal risk [74]. In the moral scenario of the old GP, we asked how acceptable participants would find it if he closed his practice because he belongs to a high-risk group due to his old age. Overall, we found high acceptance to close the practice with little variation across the three assessment periods and found that acceptance was particularly high for participants who scored higher in fear. Participants might have imagined themselves in the same situation and have evaluated the risk if the practice remains open accordingly. Moreover, we found an interaction between country and age. In the German sample, there was almost no difference based on age, but for Italians, older people expressed a lower acceptance to close down the practice. This could be explained by the overburdened health care system in Italy during the first wave. Older people in Italy might have had the fear that physicians might not work because of their own risk; and consequently, patients might not get adequate care, which would have more severe consequences for the older generation. The overburdened health care system in Italy, especially during T1, could also explain why Italians showed a lower acceptance to close the practice in T1 and T2 compared to T3 and in general compared to Germans. In the later assessment periods the situation was much more relaxed in both countries.

In another scenario, we asked participants whether they found it acceptable to stockpile medical supplies and food for personal use. Stockpiling was in general not accepted, with most people indicating that they found it reprehensible. Stockpiling has not only been described as irrational and impulsive but has also led to significant shortages for many people [52], e.g., [75]. It is particularly unethical towards disadvantaged groups because not everyone has the means to buy large stocks in advance. While the acceptability was generally low in our sample, some reasons might justify stockpiling behavior. It could be especially relevant for people who are more susceptible to the virus and people who were specifically encouraged to reduce their mobility, such as the elderly [76]. It is therefore not surprising that we found that, in T1, older people found it more acceptable to stockpile. In contrast to our findings, other studies did not find an effect of age on stockpiling [77,78,79]. Although with considerable variation across the three time periods, our study indicates that increased fear led to a higher acceptability of stockpiling. This finding supports previous reports that found a relation between stockpiling and anxiety [80], as well as stockpiling and fear [81]. In our study, we also found that higher fear led to a higher acceptability of stockpiling, with considerable variation across the three time periods.

Overall, the participants in our study favored a fair allocation of resources. In addition to asking bout stockpiling, we asked two more questions regarding the distribution of goods on a societal level. First, we asked whether a hypothetical company should be restricted from selling scarce medical products to the general public and only to medical institutions during the pandemic. Second, we asked whether this company should be able to raise prices according to the increased demand and thereby increase their profit margin.

For the first scenario, the judgments differed between T1 and T2/T3. This was additionally qualified by an interaction with age. In T1, older participants found market restrictions much more appropriate compared to younger participants. At the time, there was news about shortages of medical equipment in many parts of the world. For instance, in Italy, single-use high-filtration N-95 masks had to be used multiple times [51]. Older people as potential high-risk groups might have been more concerned that they would not be properly treated if hospitals ran out of equipment. For T2 and T3, the age difference was much smaller and did not differ between the two assessment periods. During these times, the situation regarding both regulations like lock-downs as well as shortages regarding resources was considerably more relaxed, and people might have realized that there is no need to fear shortages of medical supplies. In addition, we found an interaction between country and fear, albeit with a small effect size. While all participants favored restrictions, Germans found restrictions even more acceptable than Italians. For Italians, restrictions were especially high for people with higher fear scores. In Italy, the health care system was generally more overloaded. People might have been more undecided regarding the restrictions because of the need to protect themselves with medical supplies in a system in which they might not get access to sufficient care in medical institutions.

Regarding price gouging in terms of medical supplies, such as facemasks or disinfectants, people showed a clear tendency to restrict companies from increasing prices. At the beginning of the pandemic, shortages in medical supplies were common and many people were confronted with unreasonable prices. We found a significant effect of age on the ratings of price gouging. Older people found it less acceptable to gain profit from adjusting prices. The age differences are potentially rooted in shifts in motives and moral motivation. Several studies found that people become more communally oriented with increased age and less agentic [82,83]. Agentic individuals are defined through individual interests in the self, whereas communal motives promote the interest of others and contribute to social cohesion. Therefore, selfishly motivated profits might be seen as more reprehensible by people with communal motives.

The study of repugnant market transactions, i.e., market exchanges that evoke aversion and may be constrained or prevented by third parties [84], offers an explanation for the results concerning market regulation and price gouging. Stricter market regulations were favored by the majority of the participants in our study. This does not only show that people find it unfair to exploit shifts in demand by raising prices [85], but that the desire for regulation might be rooted in moral outrage, an emotion that motivates people to punish others for exploitive behavior [86]. Moral questions of stockpiling retained relevance in the COVID-19 pandemic, especially regarding medical resources such as vaccines. Despite calls for transnational solidarity, there were mainly profit-driven national strategies that led to high-income countries having easier access and higher availability of treatments as well as vaccines [87]. Even before vaccines became available, scholars emphasized that high-income countries have a particular (moral) responsibility to ensure a fair distribution of relevant resources e.g., [88,89]. This claim is corroborated by the low acceptability ratings in our study. Both personal and institutional stockpiling was condemned by the majority of the participants of our study.

As outlined above, most people indicated a value of 50 for the scenarios regarding the target and strictness of prevention measures. Most participants therefore favored a base level of restrictions that applied to everyone, while fewer were inclined to impose strict restrictions out of solidarity with high-risk groups. People who reported higher fear favored less targeted measures to specific groups. Interestingly, however, people who reported higher fear were also in favor of looser lockdown measures to ease the economy, which was particularly pronounced in the group of German participants. This might be explained by the psychological distress that is caused by (the fear of) unemployment (see, for example, [90]). Another study found that compliance with lockdown and social distancing measures depends mostly on people’s normative obligation and intrinsic motivation to comply for reasons other than fear [91]. While fearful people might favor more lenient lockdown measures, it does not mean that they are less willing to comply with the measures that are implemented by the government. We also found that age influenced the decision for the more strict or lenient restrictions in the opposite direction for Germans compared to Italians. In Germany, older people compared to younger people preferred stricter measures, whereas in Italy, younger people favored stricter measures. There is no straightforward explanation for these differences. Although Germany and Italy are in close proximity, they differ in a number of demographics such as age structure, which was also shown to influence, for instance, the case-fatality rate in the COVID-19 pandemic [92,93]. Younger people in Italy might have felt a higher personal risk because of the higher case-fatality rate compared to Germany. In addition, they might have also perceived a higher risk for their relatives and therefore favored stricter measures.

The moral scenarios that we investigated in this study became highly relevant at the beginning of the pandemic. As the pandemic progressed, further questions about distributive justice arose. For instance, regarding triage, other diseases that require immediate treatment (e.g., cancer) were postponed to ensure capacity for the treatment of COVID-19 patients [94]. Another example of triage concerns shortages of vaccines, which have led to the need to set criteria for the prioritization of certain populations [95]. In terms of the environmental impact of the pandemic, it has been shown that there have been short-term improvements regarding pollution, global carbon emissions, and air quality [96,97]. On a meta level, this has led to moral tradeoffs regarding the environment versus economic activities. These upcoming questions affect people worldwide and highlight the importance of studying lay peoples’ opinions about distributive justice, both within the pandemic and beyond.

## 5. Conclusions

Our study provides insights into laypeople’s moral reasoning and demonstrates changes in moral intuitions due to experiences with an ongoing pandemic. Taken together, the results of the moral scenarios in our study reflect the complexity of moral reasoning in real life. We found that the person variable nationality/country of residence, age, and fear, as well as the timely proximity to an emergency affect moral decisions. The differences between the two countries and across assessment periods show that situational differences play a role in how these moral questions are evaluated. Furthermore, the results from this study shed light on how policies are perceived by the public. Public support for guidelines can increase the accountability and legitimacy of certain measures. Humanity will have to deal with future crises, primarily the climate crisis, which will likely bring further epidemics and pandemics [98]. Learning about public opinion from this pandemic will give society a better directive to handle future crises. 

## Figures and Tables

**Table 1:** Description of the moral scenarios for all time points.

Question	Description
(i) **Triage**	In the situation of the current Corona pandemic, hospitals are currently overfilled. 1000 Patients with symptoms show up but immediate treatment is only available for 500 patients. How should patients be prioritized? 0 = Young ones and otherwise healthy ones should be prioritized as they have a higher chance of survival, so the treatment is most likely more effective. 100 = Older ones and people with pre-existing conditions should be prioritized and given health care first as they are at a higher risk of dying; however, their risk of dying even with the treatment is a lot higher.
(ii) **Stockpiling**	The pandemic hit and markets are running out of medical supplies (masks, disinfections) and canned food. How acceptable is it to stock up on medical supplies and food for you personally even though this might lead to shortages for other people? 0 = Very unacceptable 100 = Very acceptable
(iii) **Old GP**	A registered general practitioner is already 65 years old. In his medical practice, he usually treats many patients including vulnerable patient groups. He is now considering closing his practice during the pandemic as he is at high risk due to his age. How acceptable is it for him to close down the practice? 0 = Very unacceptable 100 = Very acceptable
(iv) **Price Gouging**	Should Wufa be able to raise prices for disinfectants and medical masks during the pandemic as there is an increased demand, thereby increasing its own profit margin? 0 = Definitely no 100 = Definitely yes
(v) **Market Regulation**	Wufa is a medical company specialized in the production of disinfectants and medical masks. Should they be restricted from selling their products to the public and only be able to sell to medical institutions during the pandemic? 0 = Definitely no 100 = Definitely yes
***Added moral scenarios in T2 and T3***	
(vi) **Strictness of** **Measures**	In your opinion, how strict should the measures that are taken as a result of corona (e.g., exit restriction) be? 0 = Lock-down measures should be strictly enforced to protect high-risk groups and contain the pandemic (e.g., closure of schools and day-care, no restaurant/café opening, no events) 100 = The measures should be relaxed completely in order to limit the economic losses and minimize the social impact on the population as a whole
(vii) **Target of Prevention Measures**	How targeted to a specific group do you think the protective measures to reduce corona infection (e.g., travel/exit restrictions, social distancing, compulsory masks) should be? 0 = Only people who belong to the high-risk group (older people, people with previous illnesses) should distance themselves socially and thus protect themselves from infection. 50 = There should be a basic level of restriction for all (e.g., no major events) but specific restrictions to high-risk populations 100 = The restrictions should apply to everyone

## Data Availability

Data and the code behind the analyses has been made publicly available at the Open Science Framework and can be accessed at osf.io/gpfe7 (accessed on 14 September 2022).

## Supplementary Materials

The following supporting information can be downloaded at: https://www.mdpi.com/article/10.3390/ijerph191912067/s1, Description of additional scenarios including introduction, methods, data analysis, results, and discussion; Table S1: Description of the questions assessing subjective fear, confidence, and situational factors; Table S2: Sociodemographic characteristics of the participants that were included in the analyses; Table S3: Summary of Moral Scenarios GLMMs; Table S4: Summary of Moral Scenarios Reduced GLMMs; Table S5: Summary of Additional models; Table S6: Summary of Reduced Additional models; Figure S1: Distribution of the responses for each additional scenario; Figure S2: Significant main effects and interactions for the situation assessment regarding the health care system, the economy, and personal situation; Figure S3: Significant main effects and interactions for the additional questions regarding behavior changes, confidence, and long-term consequences. References [99,100,101,102,103,104,105,106,107,108] are cited in the Supplementary Materials.
