# Supplementary material for: How German and Italian Laypeople Reason about Distributive Shortages during COVID-19
Source: Int J Environ Res Public Health. 2022 Sep 23;19(19):12067. doi: 10.3390/ijerph191912067 (PMC9566015; doi:10.3390/ijerph191912067)
Supplement: Supplementary file 1 [file ijerph-19-12067-s001.zip › ijerph-1865253-supplementary.pdf]

Supplementary Material

How German and Italian laypeople reason about distributive shortages during COVID-19

Ronja Demel [1,2,3*], Francesco Grassi [1], Yasaman Rafiee [1,3], Michael R Waldmann[1,3], and Annekathrin Schacht [1,3]

1 Institute of Psychology, Georg-August University Goettingen, Germany
2 Institute of Psychology, Humboldt-University Berlin, Germany
3 Leibniz ScienceCampus "Primate Cognition", Goettingen, Germany
* Correspondence: ronja.demel@hu-berlin.de

Supplementary Material 1: Additional Scenarios

Introduction

In addition to the moral scenarios that were posed in the present study, we asked several questions to gain a better understanding about participants' impression of the situation across the three time periods. These questions include a confidence rating, a situation assessment concerning the health system, the economy, their personal life, and their job situation as well as an estimation of the long-term effects of the pandemic for their personal life and the society. For the fear assessment, we used an assessment adapted from the Fear of Coronavirus-19 scale [1], which was widely used in studies on the COVID-19 pandemic and therefore allows to compare our data to the data of other studies.

Methods

Across all three time periods, participants were asked how confident they felt about the corona-virus situation. In T2 and T3, participants were asked for an individual situation assessment concerning the health system, the economy, their personal life, and their current job situation. Also in T2 and T3, the fear assessment was refined by using a total of 10 questions based on the *Fear of Coronavirus-19 scale* [1]. Since this fear measure has been highly used during the COVID-19 pandemic, the data from our study can be compared to other studies. However, for the purpose of this study, only the three fear questions that were assessed in all assessment periods were considered as a measure of COVID-19 related fear. Participants were further asked about their behavior changes due to the corona pandemic in accordance with Harper et al. [2]. Finally, T3 included two additional questions that assessed the personal estimation of the long-term effects of the pandemic on participants' personal life and on the society. Please see Table S1 for the description of all additional scenarios.

Table S1

Description of the questions assessing subjective fear, confidence, and situational factors

Question	Description
Confidence	How confident do you feel about the situation concerning the corona virus?<br>0 = not at all confident<br>100 = very confident
Fear 1	How afraid are you of being infected?<br>0 = not afraid<br>100 = very afraid
Fear of death for self or closely related persons	How afraid are you of you or someone close to you dying due to the corona virus?<br>0 = not afraid<br>100 = very afraid
Health system/ Economy/ Personal life	Do you think the circumstances concerning the health system in your country/ the economy in your country//your personal life driven by the pandemic are better or worse compared to filling out the first part of the questionnaire (mid-March)?<br>0= much worse than before<br>50= same as before<br>100= much better than before
"Job Situation (1)"	Was your job/study situation negatively affected by the corona pandemic?<br>Yes<br>No
"Job Situation (2)"	How is your work/study situation at the moment<br>Same as before<br>In the home office<br>Reduced or no work hours with reduced salary<br>Unpaid vacation<br>Paid vacation<br>Job termination due to corona virus other
"Behavior Change"	How much did you change your protective behavior (e.g. hand washing, working from home, social distancing, travels, grocery shopping) compared to when you filled out the first part of the questionnaire (mid-March)?<br>0 = has decreased<br>100 = has increased
Long-Term Personal/Society	Do you think the pandemic will have long-term consequences for your personal life/the society?<br>0 = no consequences<br>100 = significant consequences

Data Analysis

Generalized Linear Mixed Models GLMM; [3] with Gaussian error structure were used to model the response to each of the situation assessment questions, as well as the questions on behavior changes, and confidence. These models included country, assessment period, and their interaction as fixed effects. Covariates age and fear, their interaction, as well as the interaction of fear and country, fear and assessment period, age and country, and age and assessment period, were also included as fixed effects. Participant was included as a random intercept effect.

The overall effect of fixed effects country and assessment period on the rating to the situation assessment questions was tested by comparing each full model with a null model lacking the two fixed effects, using a likelihood ratio test. Similarly, the overall effect of country, assessment period, and fear on the rating of the behavior change and the confidence questions was tested by comparing each model with a null model lacking the three fixed effects. Moreover, a linear model was used to model the response to each of the long-term effects questions. The models included country, fear, and their interaction as fixed effects. Age, as well as its interaction with country and fear, was also included as fixed effects. For all models, the main effect of predictors not involved in any significant interaction was tested by fitting a reduced model lacking those interactions.

Results

The distributions of the response to each question regarding the situation assessment, behavior changes, and confidence are shown in **Figure S1**. All the assumptions of the models, including collinearity, as well as normality and homogeneity of the residuals, were met in each model. Summaries of the models can be found in Table S5 for the full models, and Table S6 for the respective reduced models.

Figure S1
Distribution of the responses for each additional scenario

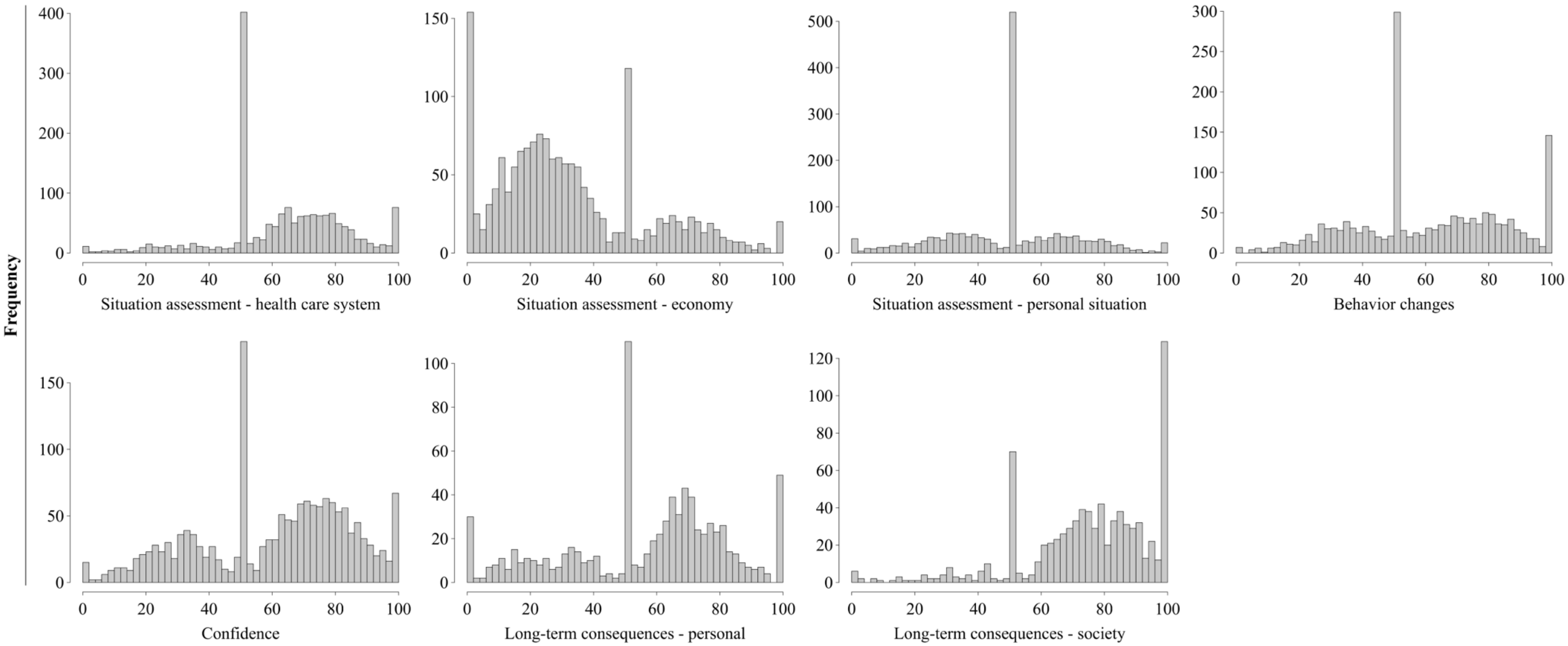

Situation assessment

In T2 and T3, participants were asked to evaluate whether they thought that the situation regarding the health care system, the economy, and their personal life had gotten better or worse compared to the beginning of the pandemic (T1). Regarding the health care system, both Italians and Germans found that the situation in T2 and T3 was improved (M_{T2} = 61.43, SD_{T2} = 19.85; M_{T3} = 65.33, SD_{T3} = 19.18). In addition, they indicated a stronger improvement of the situation between T3 and T1 than between T2 and T1, with this difference being stronger in Italians (χ^2 = 4.52, *df* = 1, *p* = .034; **Figure S2.A**). A fear by nationality interaction indicated that at lower fear levels, Italians evaluated the public health situation slightly better than Germans, while at higher fear levels this pattern was reversed (χ^2 = 3.93, *df* = 1, *p* = .047; **Figure S2.B**). Finally, the interaction of fear and age was significant (χ^2 = 7.80, *df* = 1, *p* = .005), indicating decreasing situation judgments with higher age at lower levels of fear, while at higher fear levels, older people indicated that situation of the health care system as better (**Figure S2.C**).

Overall, participants assessed the economic situation as worse in T2 and T3 compared to T1 (M_{T2} = 28.75, SD_{T2} = 21.98; M_{T3} = 37.42, SD_{T3} = 24.45). Italians showed lower ratings of the economic situation than Germans (β = -10.23, *SE* = 1.36, *p* < .001; **Figure S2.E**). The interaction of assessment period and age was significant (χ^2 = 5.56, *df* = 1, *p* = .018). While age differences were absent at T2, older compared to younger participants judged the economy as worse at T3 (**Figure S2.D**). Participants with higher fear judged the economic situation slightly better than participants with lower fear (β = 1.54, *SE* = 0.60, *p* = .010; **Figure S2.F**).

There was no main effect of assessment period on participants' judgments of their personal situation, i.e., participants did not indicate any changes in either T2 or T3 compared to T1 (M_{T2} = 46.36, SD_{T2} = 19.92; M_{T3} = 52.85, SD_{T3} = 20.28). However, similarly to their rating of the economic situation, older participants indicated that their situation worsened in T3 but not in T2 compared to T1, as indicated by an interaction of assessment period and age (χ^2 = 7.23, *df* = 1, *p* = .007; **Figure S2.G**). In addition, and again similarly to the economic situation, the personal situation was rated as worse by Italians compared to Germans (β = -2.61, *SE* = 1.20, *p* = .030; **Figure S2.H**).

Behavior changes

Participants indicated that they slightly expanded their protective behavior from T1 to T2 (*M* = 66.88, *SD* = 22.02) and from T1 to T3 (*M*s = 55.01, *SD*s = 23.92). Protective behavior was also higher in T3 compared to T2, with a higher increase for Germans compared to Italians (χ^2 = 8.32, *df* = 1, *p* = .004; **Figure S3.A**). In addition, older participants reported an increase in protective behaviors compared to younger participants, independently of the country and assessment period (β = 4.58, *SE* = 0.66, *p* < .001; **Figure S3.C**). The interaction of assessment period and fear was also significant (χ^2 = 8.55, *df* = 1, *p* = .003). Higher fear led to an overall increase in protective behavior, with a particular increase in T2 compared to T3 (**Figure S3.B**).

Confidence

Participants were asked how confident they felt about the current situation during the pandemic. Higher fear led to lower confidence ratings, which was amplified for Italians compared to Germans ($\chi^2 = 7.46$, $df = 1$, $p = .006$; **Figure S3.D**). In addition, a slight increase in confidence at T3 compared to T2 could be observed for participants from both countries ($\beta = 2.57$, $SE = 0.86$, $p = .003$; **Figure S3.E**).

Long-term consequences

Participants were asked whether they believed that the pandemic would lead to long-term consequences for them personality and for society. Participants generally expected stronger consequences for society ($M = 75.14$, $SD = 20.53$) as a whole than for them personally ($M = 57.11$, $SD = 25.60$). However, the same predictors significantly impacted the rating scores in both items: Younger Italians expected more consequences compared to young Germans, while for older participants, this pattern was the opposite, with older Germans expecting more consequences than older Italians ($\beta_{personal} = -6.27$, $SE_{personal} = 1.92$, $p_{personal} = .001$; $\beta_{society} = -4.32$, $SE_{society} = 1.55$, $p_{society} = .005$, **Figure S3.F,H**). In addition, higher fear led to the expectation of more long-term consequences ($\beta_{personal} = 6.01$, $SE_{personal} = 0.87$, $p_{personal} < .001$; $\beta_{society} = 3.90$, $SE_{society} = 0.70$, $p_{society} < .001$; **Figure S3.G, I**).

Figure S2

Significant main effects and interactions for the situation assessment regarding the health care system, the economy, and personal situation.

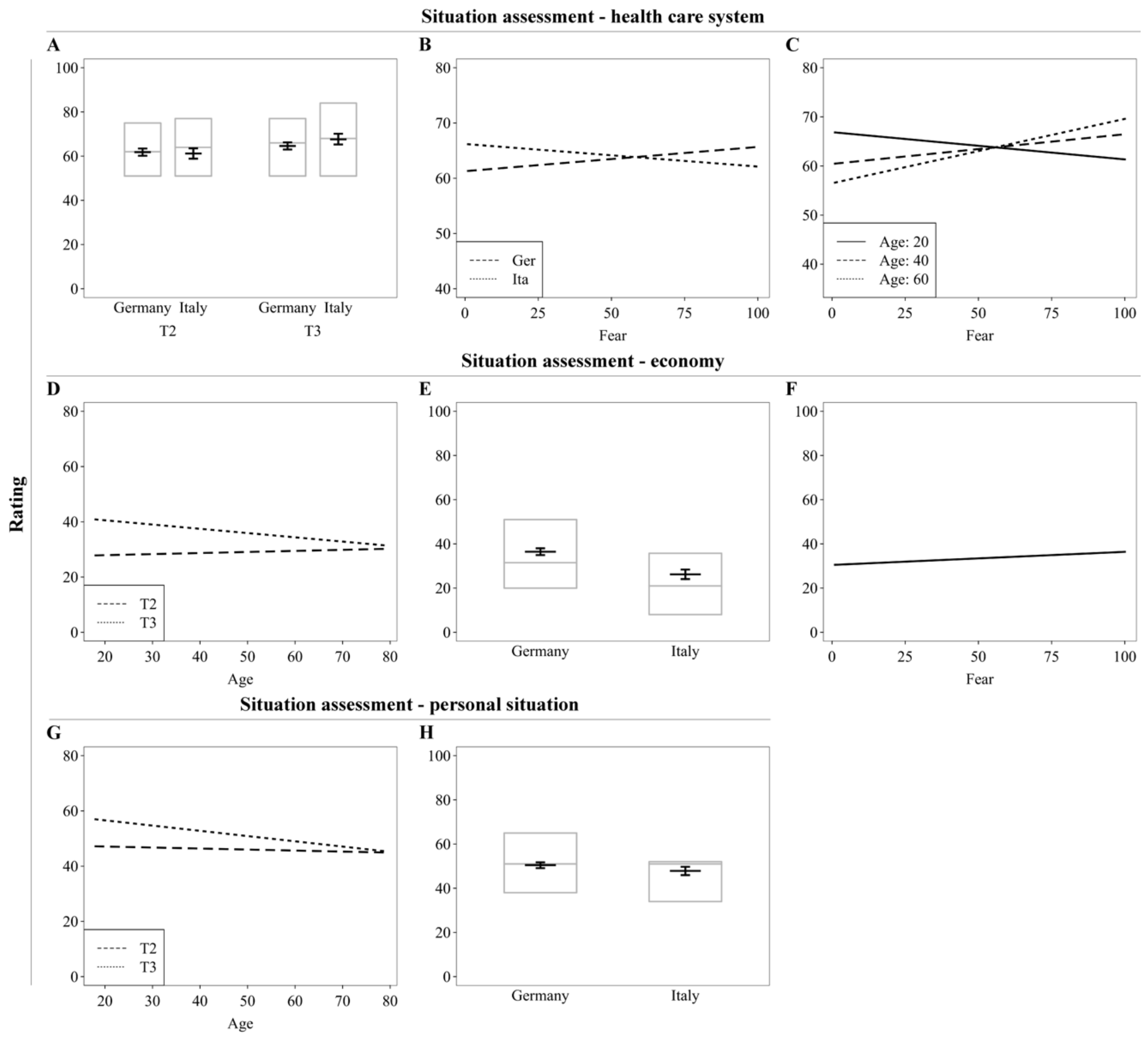

Figure S3

Significant main effects and interactions for the additional questions regarding behavior changes, confidence, and long-term consequences.

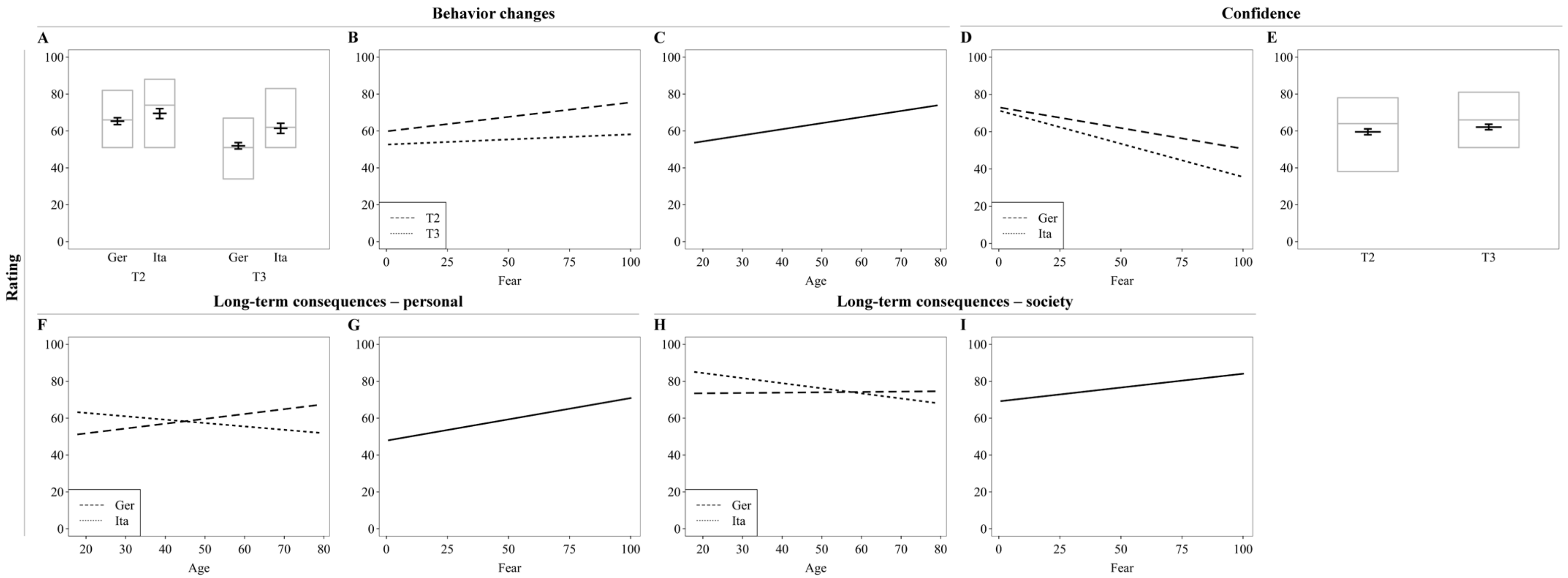

Discussion

In addition to the moral scenarios that are described in the main article, we asked about other aspects of the health care system, the economy, participants' personal lives in T2 and T3, how participants changed their behavior in T2 and T3, and what long term-term consequences they expected for them personally and for the society in T3. Taken together, these measures provide a good overview of how the situation in the two countries changed over time and how participants perceived the current situation and adapted their behavior accordingly.

We assessed how participants felt about their situation at T2 and T3 in comparison to T1 to get an impression of whether there are differences between Germany and Italy and between the assessment periods. Regarding the health care system, there was a slight improvement from T2 to T3. While for Italians, higher fear led to rate the situation of the health care system as worse, it was exactly the opposite for Germans. Regarding the economy, participants indicated that the situation deteriorated throughout our assessment periods but was less precarious in T3 compared to T2. Compared to Germans, Italians rated the impact of the corona pandemic on the economy as worse. For the personal situation, there was no difference between the countries or assessment periods but older people indicated that their situation became worse compared to younger people who expressed that the situation for them was the same as before. The ratings show that both the health care system and the economy were burdened by the long-lasting consequences of the corona pandemic. This is also reflected by the high utilization rates of the hospitals [4], as well as the drop in GDP of the two countries [5]. The higher fear scores for Italians regarding the health care system probably reflect the overburdening of the health care system in Italy.

Apart from a situation assessment, we asked whether participants' behavior changed in T2 and T3. Following a study by Harper, Satchell, Fido, and Latzman [2] we assessed whether fear, assessment period, country, and age influenced behavior changes, such as hand washing, working from home, social distancing, travels, grocery shopping. Our results showed that there was an overall increase of behavior changes in T2 and T3 compared to T1, but this increase was lower in T3 compared to T2. Thus, the behavior changes were probably adaptive to the situation, which can change even in the course of a few months. In addition, higher behavior changes were reported by Italians compared to Germans, in particular in the elderly and people with higher fear. In the case of the corona pandemic, fear may have served as a protective function as it leads to adopting relevant behavior changes to mitigate the risk of a virus transmission, which was also reported in other studies [2], [6], [7].

As another question to get a better insight on how participants felt about the current corona situation, we asked them how confident they felt. We found that people with higher fear reported significantly less confidence, especially in the Italian sample. Interestingly, the opposing influence of fear on behavior changes and confidence contradicts some findings of other studies. For instance, it was found that self-confidence and confidence in governmental and medical institutions sometimes

tend to increase the adoption of prevention measures [8]–[10]. However, consistent with our results, anxiety was negatively correlated with confidence [11]. Another study also found that preventive measures were predicted by self-reported worry [12]. Thereby, fear can be a prominent predictor of preventive measures. Future studies need to clarify how this discrepancy between the findings can be explained.

As a final question in T3, we asked participants whether they expected long-term consequences for their personal life and society. Overall, the majority of participants indicated that they see substantial consequences for their personal lives but especially for society as a whole. In both questions, Italians predicted larger consequences compared to Germans; however, older ones generally expressed to expect fewer long-term consequences. Participants with higher fear also saw more long-term consequences for society and their personal lives. Other studies showed that several factors related to the corona pandemic, such as consequences of a COVID-19 infection [13], or school closures due to lockdowns [14] have long-lasting consequences on the social, economic and psychological well-being of people.

References

1. Ahorsu, D. K.; Lin, C. Y.; Imani, V.; Saffari, M.; Griffiths, M. D.; Pakpour, A. H. The Fear of COVID-19 Scale: Development and Initial Validation. *International Journal of Mental Health and Addiction*, **2020**. https://doi.org/10.1007/s11469-020-00270-8.
2. Harper, C. A.; Satchell, L. P.; Fido, D.; Latzman, R. D. Functional Fear Predicts Public Health Compliance in the COVID-19 Pandemic. *International Journal of Mental Health and Addiction*, **2020**. https://doi.org/10.1007/s11469-020-00281-5.
3. Baayen, R. H.; Davidson, D. J.; Bates, D. M. Mixed-Effects Modeling with Crossed Random Effects for Subjects and Items. *Journal of Memory and Language*, **2008**, *59* (4), 390–412. https://doi.org/10.1016/j.jml.2007.12.005.
4. Pecoraro, F.; Luzi, D.; Clemente, F. The Efficiency in the Ordinary Hospital Bed Management: A Comparative Analysis in Four European Countries before the COVID-19 Outbreak. *PLoS ONE*, **2021**, *16* (3 March), 1–18. https://doi.org/10.1371/journal.pone.0248867.
5. McKibbin, W.; Fernando, R. The Economic Impact of COVID-19. In *Economics in the Time of COVID-19*; Baldwin, R., Mauro, B. W. di, Eds.; 2020; pp 45–51.
6. Winter, T.; Riordan, B. C.; Pakpour, A. H.; Griffiths, M. D.; Mason, A.; Poulgrain, J. W.; Scarf, D. Evaluation of the English Version of the Fear of COVID-19 Scale and Its Relationship with Behavior Change and Political Beliefs. *International Journal of Mental Health and Addiction*, **2020**. https://doi.org/10.1007/s11469-020-00342-9.
7. Yıldırım, M.; Geçer, E.; Akgül, Ö. The Impacts of Vulnerability, Perceived Risk, and Fear on Preventive Behaviours against COVID-19. *Psychology, Health & Medicine*, **2021**, *26* (1), 35–43. https://doi.org/10.1080/13548506.2020.1776891.
8. Storopoli, J.; Braga da Silva Neto, W. L.; Mesch, G. S. Confidence in Social Institutions, Perceived Vulnerability and the Adoption of Recommended Protective Behaviors in Brazil during the COVID-19 Pandemic. *Social Science and Medicine*, **2020**, *265*, 113477. https://doi.org/10.1016/j.socscimed.2020.113477.
9. Bargain, O.; Aminjonov, U. Trust and Compliance to Public Health Policies in Times of COVID-19. *Journal of Public Economics*, **2020**, *192* (13205). https://doi.org/10.1016/j.jpubeco.2020.104316.
10. Chan, H. F.; Brumpton, M.; Macintyre, A.; Arapoc, J.; Savage, D. A.; Skali, A.; Stadelmann, D.; Torgler, B. How Confidence in Health Care Systems Affects Mobility and Compliance during the COVID-19 Pandemic. *PLoS ONE*, **2020**, *15* (10 October), 1–18. https://doi.org/10.1371/journal.pone.0240644.

11. Cui, S.; Jiang, Y.; Shi, Q.; Zhang, L.; Kong, D.; Qian, M.; Chu, J. Impact of Covid-19 on Anxiety, Stress, and Coping Styles in Nurses in Emergency Departments and Fever Clinics: A Cross-Sectional Survey. *Risk Management and Healthcare Policy*, **2021**, *14*, 585–594. https://doi.org/10.2147/RMHP.S289782.
12. Sobkow, A.; Zaleskiewicz, T.; Petrova, D.; Garcia-Retamero, R.; Traczyk, J. Worry, Risk Perception, and Controllability Predict Intentions Toward COVID-19 Preventive Behaviors. *Frontiers in Psychology*, **2020**, *11* (November), 1–15. https://doi.org/10.3389/fpsyg.2020.582720.
13. del Rio, C.; Collins, L. F.; Malani, P. Long-Term Health Consequences of COVID-19. *JAMA*, **2020**, *324* (17), 1723. https://doi.org/10.1001/jama.2020.19719.
14. Fuchs-Schündeln, N.; Krueger, D.; Ludwig, A.; Popova, I. *The Long-Term Distributional and Welfare Effects of Covid-19 School Closures*; Cambridge, MA, 2020; Vol. 53. https://doi.org/10.3386/w27773.

Supplementary Material 2

Table S2

Sociodemographic characteristics of the participants that were included in the analyses

	Germany (N=551)	Italy (N=269)	Total (N=820)
Age (Years)			
Mean (SD)	31.0 (11.9)	35.5 (12.4)	32.5 (12.3)
Median [Min, Max]	27.0 [18.0, 79.0]	30.0 [18.0, 78.0]	28.0 [18.0, 79.0]
Gender			
Male	150 (27.2%)	77 (28.6%)	227 (27.7%)
Female	398 (72.2%)	192 (71.4%)	590 (72.0%)
Other	3 (0.5%)	0 (0%)	3 (0.4%)
Occupation			
Student	223 (40.5%)	64 (23.8%)	287 (35.0%)
Edu/Res	94 (17.1%)	56 (20.8%)	150 (18.3%)
Art/Entert./Sport/Media	21 (3.8%)	5 (1.9%)	26 (3.2%)
Health care	41 (7.4%)	43 (16.0%)	84 (10.2%)
Military	0 (0%)	0 (0%)	0 (0%)
Comm./Social serv./Politics	42 (7.6%)	7 (2.6%)	49 (6.0%)
Finance/Business	12 (2.2%)	10 (3.7%)	22 (2.7%)
Industry	21 (3.8%)	9 (3.3%)	30 (3.7%)
Services	27 (4.9%)	13 (4.8%)	40 (4.9%)
Transport	3 (0.5%)	2 (0.7%)	5 (0.6%)
Cleaning	1 (0.2%)	2 (0.7%)	3 (0.4%)
Other	56 (10.2%)	47 (17.5%)	103 (12.6%)
Unemployed	10 (1.8%)	11 (4.1%)	21 (2.6%)
Highest educational level			
None	1 (0.2%)	0 (0%)	1 (0.1%)
Primary	2 (0.4%)	2 (0.7%)	4 (0.5%)
Secondary	262 (47.5%)	52 (19.3%)	314 (38.3%)
Bachelor	142 (25.8%)	48 (17.8%)	190 (23.2%)
Master	89 (16.2%)	121 (45.0%)	210 (25.6%)
PhD	20 (3.6%)	43 (16.0%)	63 (7.7%)
Other	35 (6.4%)	3 (1.1%)	38 (4.6%)
Pre-existing condition			
Yes	104 (18.9%)	32 (11.9%)	136 (16.6%)

Table S2

Sociodemographic characteristics of the participants that were included in the analyses

	Germany (N=551)	Italy (N=269)	Total (N=820)
No	447 (81.1%)	237 (88.1%)	684 (83.4%)
Infection status			
Self	0 (0%)	1 (0.4%)	1 (0.1%)
Known person	31 (5.6%)	34 (12.6%)	65 (7.9%)
No	520 (94.4%)	234 (87.0%)	754 (92.0%)
Religiousness			
Mean (SD)	21.8 (29.3)	31.1 (32.6)	24.9 (30.8)
Median [Min, Max]	5.00 [1.00, 100]	18.0 [1.00, 100]	8.00 [1.00, 100]

Table S3. Summary of Moral Scenarios GLMMs

	Old GP					Stockpiling					Market Regulation					Price Gouging				
Predictors	*Estimates*	*std. Error*	*CI*	*z value*	*p*	*Estimates*	*std. Error*	*CI*	*z value*	*p*	*Estimates*	*std. Error*	*CI*	*z value*	*p*	*Estimates*	*std. Error*	*CI*	*z value*	*p*
intercept	0.82	0.06	0.68 – 0.92		(1)	-1.39	0.05	-1.49 – -1.28		(1)	1.11	0.06	0.99 – 1.22		(1)	-2.00	0.06	-2.12 – -1.89		(1)
country (Italy)(2)	-0.38	0.12	-0.59 – -0.13	-3.21	0.001	0.02	0.10	-0.17 – 0.22	0.18	0.860	-0.78	0.11	-0.97 – -0.58	-7.33	<0.001	-0.14	0.10	-0.34 – 0.05	-1.36	0.173
assessment period (T2)(3)	0.20	0.07	0.08 – 0.33	3.10	0.002	0.05	0.06	-0.07 – 0.16	0.80	0.425	-0.59	0.07	-0.71 – -0.45	-8.97	<0.001	0.30	0.06	0.19 – 0.42	5.15	<0.001
assessment period (T3)(3)	0.02	0.07	-0.11 – 0.15	0.27	0.786	0.05	0.06	-0.07 – 0.17	0.88	0.381	-0.59	0.07	-0.71 – -0.46	-8.89	<0.001	0.29	0.06	0.17 – 0.40	4.82	<0.001
age(4)	0.00	0.06	-0.11 – 0.12	-0.01	0.995	0.14	0.05	0.04 – 0.22	2.82	0.005	0.27	0.05	0.16 – 0.36	4.92	<0.001	-0.10	0.05	-0.21 – -0.01	-2.00	0.046
fear(5)	0.17	0.06	0.06 – 0.26	2.98	0.003	0.07	0.05	-0.02 – 0.17	1.42	0.157	-0.05	0.05	-0.15 – 0.06	-0.90	0.371	-0.04	0.05	-0.14 – 0.05	-0.91	0.363
country (Italy)*assessment period (T2)	0.11	0.12	-0.12 – 0.35	0.94	0.349	0.04	0.10	-0.16 – 0.25	0.38	0.701	-0.19	0.12	-0.44 – 0.04	-1.63	0.103	-0.02	0.11	-0.24 – 0.19	-0.22	0.824
country (Italy)*assessment period (T3)	0.35	0.12	0.11 – 0.56	2.87	0.004	0.15	0.10	-0.06 – 0.35	1.44	0.151	0.01	0.12	-0.21 – 0.24	0.12	0.903	-0.01	0.11	-0.23 – 0.21	-0.11	0.912
assessment period (T2)*age	0.08	0.06	-0.02 – 0.19	1.48	0.139	0.02	0.05	-0.07 – 0.12	0.36	0.720	-0.16	0.05	-0.26 – -0.05	-2.96	0.003	0.03	0.05	-0.08 – 0.14	0.58	0.561
assessment period (T3)*age	0.04	0.06	-0.07 – 0.14	0.66	0.513	0.08	0.05	-0.01 – 0.18	1.74	0.082	-0.20	0.06	-0.30 – -0.08	-3.54	<0.001	-0.03	0.05	-0.13 – 0.08	-0.57	0.568
country (Italy)*age	-0.30	0.09	-0.47 – -0.12	-3.27	0.001	-0.05	0.07	-0.18 – 0.10	-0.64	0.522	0.02	0.08	-0.13 – 0.17	0.27	0.785	0.10	0.08	-0.05 – 0.25	1.38	0.169
assessment period (T2)*fear	-0.04	0.06	-0.16 – 0.07	-0.71	0.477	0.01	0.05	-0.10 – 0.11	0.25	0.805	-0.05	0.06	-0.16 – 0.05	-0.87	0.382	0.05	0.05	-0.05 – 0.15	1.01	0.313
assessment period (T3)*fear	0.01	0.06	-0.10 – 0.12	0.17	0.866	0.12	0.05	0.01 – 0.21	2.25	0.024	0.01	0.06	-0.10 – 0.12	0.18	0.854	0.07	0.05	-0.04 – 0.16	1.26	0.206
country (Italy)*fear	0.00	0.07	-0.12 – 0.15	0.02	0.984	0.05	0.06	-0.07 – 0.17	0.87	0.383	0.18	0.07	0.06 – 0.32	2.55	0.011	-0.08	0.06	-0.19 – 0.04	-1.31	0.191

(1) not shown because of being of very limited interpretability
(2) comparison with the reference level (Germany)
(3) comparisons with the reference level (T1)
(4) log- and z-transformed to mean of zero and a standard deviation of one
(5) z-transformed to mean of zero and a standard deviation of one

Table S3. (cont.) Summary of Moral Scenarios GLMMs

	**Triage**					**Target of Prevention Measures**					**Strictness of Measures**				
Predictors	*Estimates*	*std. Error*	*CI*	*z value*	*p*	*Estimates*	*std. Error*	*CI*	*z value*	*p*	*Estimates*	*std. Error*	*CI*	*z value*	*p*
intercept	0.95	0.06	0.82 – 1.07		(1)	1.02	0.06	0.88 – 1.12		(1)	-0.44	0.05	-0.54 – -0.33		(1)
country (Italy)(2)	-0.87	0.12	-1.09 – -0.63	-7.50	<0.001	-0.16	0.11	-0.38 – 0.06	-1.40	0.161	0.32	0.10	0.12 – 0.51	3.32	0.001
assessment period (T2)(3)	-0.65	0.07	-0.78 – -0.52	-9.82	<0.001										
assessment period (T3)(3)	-0.64	0.07	-0.77 – -0.52	-9.64	<0.001	-0.01	0.06	-0.14 – 0.10	-0.24	0.813	0.12	0.06	0.01 – 0.24	2.03	0.042
age(4)	-0.01	0.06	-0.12 – 0.10	-0.17	0.867	0.07	0.06	-0.05 – 0.19	1.18	0.240	0.13	0.05	0.03 – 0.23	2.55	0.011
fear(5)	0.02	0.05	-0.09 – 0.13	0.38	0.708	0.31	0.06	0.19 – 0.41	5.53	<0.001	-0.37	0.05	-0.46 – -0.28	-7.59	<0.001
country (Italy)*assessment period (T2)	0.82	0.12	0.58 – 1.05	6.91	<0.001										
country (Italy)*assessment period (T3)	0.65	0.12	0.41 – 0.87	5.41	<0.001	-0.08	0.11	-0.30 – 0.13	-0.68	0.496	-0.01	0.10	-0.20 – 0.19	-0.09	0.925
assessment period (T2)*age	-0.02	0.06	-0.13 – 0.08	-0.35	0.724										
assessment period (T3)*age	0.09	0.05	-0.01 – 0.20	1.68	0.093	-0.03	0.05	-0.12 – 0.08	-0.47	0.636	-0.03	0.05	-0.13 – 0.06	-0.66	0.509
country (Italy)*age	0.05	0.09	-0.13 – 0.22	0.52	0.602	0.06	0.10	-0.12 – 0.25	0.64	0.524	-0.21	0.08	-0.37 – -0.05	-2.57	0.010
assessment period (T2)*fear	0.09	0.06	-0.03 – 0.20	1.56	0.118										
assessment period (T3)*fear	0.03	0.06	-0.09 – 0.15	0.47	0.635	-0.04	0.05	-0.15 – 0.06	-0.78	0.437	-0.02	0.05	-0.11 – 0.08	-0.41	0.684
country (Italy)*fear	-0.01	0.07	-0.15 – 0.13	-0.15	0.882	-0.04	0.08	-0.18 – 0.13	-0.46	0.646	0.21	0.07	0.07 – 0.34	2.94	0.003

(1) not shown because of being of very limited interpretability
(2) comparison with the reference level (Germany)
(3) comparisons with the reference level (T1)
(4) log- and z-transformed to mean of zero and a standard deviation of one
(5) z-transformed to mean of zero and a standard deviation of one

Table S4. Summary of Moral Scenarios Reduced GLMMs

	Old GP					Stockpiling					Price Gouging					Triage				
Predictors	*Estimates*	*std. Error*	*CI*	*z value*	*p*	*Estimates*	*std. Error*	*CI*	*z value*	*p*	*Estimates*	*std. Error*	*CI*	*z value*	*p*	*Estimates*	*std. Error*	*CI*	*z value*	*p*
intercept	0.82	0.06	0.69 – 0.94		[1]	-1.40	0.05	-1.49 – -1.30		[1]	-2.01	0.06	-2.11 – -1.90		[1]	0.96	0.06	0.81 – 1.07		[1]
country (Italy)[2]	-0.39	0.11	-0.60 – -0.17	-3.42	0.001	0.08	0.07	-0.06 – 0.22	1.18	0.239	-0.15	0.08	-0.29 – 0.01	-1.95	0.051	-0.89	0.11	-1.11 – -0.68	-8.03	<0.001
assessment period (T2)[3]	0.19	0.06	0.06 – 0.31	2.98	0.003	0.06	0.05	-0.03 – 0.14	1.16	0.246	0.31	0.05	0.21 – 0.40	6.34	<0.001	-0.65	0.06	-0.77 – -0.51	-9.94	<0.001
assessment period (T3)[3]	0.01	0.06	-0.12 – 0.14	0.15	0.881	0.10	0.05	0.01 – 0.19	2.06	0.040	0.29	0.05	0.20 – 0.38	5.99	<0.001	-0.66	0.07	-0.79 – -0.53	-10.02	<0.001
age[4]	0.04	0.05	-0.06 – 0.13	0.75	0.454	0.16	0.03	0.09 – 0.22	4.72	<0.001	-0.07	0.04	-0.14 – 0.00	-2.08	0.037	0.03	0.04	-0.05 – 0.11	0.68	0.496
fear[5]	0.15	0.04	0.08 – 0.22	4.44	<0.001	0.07	0.04	-0.01 – 0.15	1.70	0.090	-0.03	0.03	-0.08 – 0.03	-0.93	0.351	0.05	0.03	-0.01 – 0.12	1.62	0.106

Note: no reduced model was fitted for the moral scenario Market Regulation since all predictors were in at least one significant interaction.

[1] not shown because of being of very limited interpretability
[2] comparison with the reference level (Germany)
[3] comparisons with the reference level (T1)
[4] log- and z-transformed to mean of zero and a standard deviation of one
[5] z-transformed to mean of zero and a standard deviation of one

Table S4. (cont.) Summary of Moral Scenarios Reduced GLMMs

	Target of Prevention Measures					Strictness of Measures				
Predictors	*Estimates*	*std. Error*	*CI*	*z value*	*p*	*Estimates*	*std. Error*	*CI*	*z value*	*p*
intercept	1.04	0.06	0.91 – 1.14		[1]	-0.44	0.05	-0.53 – -0.34		[1]
country (Italy)[2]	-0.19	0.10	-0.38 – 0.00	-1.95	0.051	0.32	0.08	0.15 – 0.47	3.91	<0.001
assessment period (T2)[3]										
assessment period (T3)[3]	-0.04	0.05	-0.14 – 0.06	-0.75	0.451	0.12	0.05	0.02 – 0.21	2.43	0.015
age[4]	0.08	0.04	-0.01 – 0.15	1.73	0.084	0.12	0.04	0.03 – 0.20	2.54	0.011
fear[5]	0.28	0.04	0.19 – 0.34	7.10	<0.001	-0.38	0.04	-0.46 – -0.30	-9.12	<0.001

[1] not shown because of being of very limited interpretability
[2] comparison with the reference level (Germany)
[3] comparisons with the reference level (T1)

[4] log- and z-transformed to mean of zero and a standard deviation of one
[5] z-transformed to mean of zero and a standard deviation of one

Table S5. Summary of Additional models

	Sit. Assessment - Health					Sit. Assessment - Economy					Sit. Assessment - Personal					Behavior Changes				
Predictors	*Estimates*	*std. Error*	*CI*	*t value*	*p*	*Estimates*	*std. Error*	*CI*	*t value*	*p*	*Estimates*	*std. Error*	*CI*	*t value*	*p*	*Estimates*	*std. Error*	*CI*	*t value*	*p*
intercept	61.82	0.83	60.18 – 63.44		(1)	31.6	0.97	29.73 – 33.65		(1)	47.68	0.86	45.95 – 49.40		(1)	65.35	0.94	63.59 – 67.05		(1)
country (Italy)(2)	-0.63	1.49	-3.65 – 2.03	-0.42	0.673	-8.94	1.73	-12.32 – -5.79	-5.14	<0.001	-3.92	1.53	-6.85 – -0.76	-2.55	0.011	4.54	1.68	1.44 – 7.59	2.69	0.007
assessment period (T3)(3)	2.78	0.96	0.90 – 4.71	2.87	0.004	9.77	1.19	7.41 – 12.07	8.22	<0.001	5.60	1.04	3.60 – 7.64	5.35	<0.001	-13.34	1.02	-15.35 – -11.56	-13.00	<0.001
age(4)	0.44	0.79	-1.10 – 1.99	0.56	0.578	0.22	0.91	-1.57 – 2.11	0.24	0.810	-0.31	0.81	-1.88 – 1.24	-0.38	0.706	4.98	0.90	3.21 – 6.66	5.54	<0.001
fear(5)	1.87	0.76	0.41 – 3.34	2.44	0.015	2.69	0.90	0.97 – 4.43	2.99	0.003	-0.34	0.79	-1.84 – 1.11	-0.43	0.670	3.36	0.85	1.66 – 4.95	3.94	<0.001
country (Italy)*assessment period (T3)	3.64	1.71	0.32 – 6.81	2.12	0.034	-2.90	2.10	-6.91 – 1.30	-1.38	0.169	2.60	1.85	-1.14 – 5.94	1.40	0.162	5.25	1.82	1.74 – 8.60	2.88	0.004
assessment period (T3)*age	-1.33	0.80	-2.98 – 0.18	-1.65	0.099	-2.34	0.99	-4.38 – -0.46	-2.36	0.019	-2.35	0.87	-4.09 – -0.58	-2.69	0.007	0.59	0.85	-1.02 – 2.16	0.69	0.492
country (Italy)*age	-1.35	1.22	-3.60 – 1.24	-1.10	0.272	0.77	1.38	-2.18 – 3.16	0.56	0.578	-0.24	1.22	-2.63 – 1.98	-0.20	0.844	-2.46	1.42	-5.15 – 0.40	-1.72	0.085
assessment period (T3)*fear	-1.45	0.82	-3.04 – 0.22	-1.77	0.077	-1.21	1.00	-3.25 – 0.74	-1.21	0.227	-0.56	0.88	-2.23 – 1.15	-0.64	0.524	-2.56	0.87	-4.25 – -0.89	-2.93	0.004
country (Italy)*fear	-2.20	1.11	-4.31 – 0.10	-1.98	0.048	-1.81	1.28	-4.45 – 0.75	-1.41	0.159	0.08	1.13	-2.13 – 2.31	0.07	0.941	2.23	1.25	-0.28 – 4.55	1.77	0.077
age*fear	1.47	0.53	0.53 – 2.55	2.79	0.005	0.96	0.61	-0.26 – 2.20	1.56	0.119	0.28	0.54	-0.68 – 1.29	0.52	0.602	-0.47	0.59	-1.67 – 0.67	-0.79	0.432

(1) not shown because of being of very limited interpretability
(2) comparison with the reference level (Germany)
(3) comparisons with the reference level (T1)
(4) log- and z-transformed to mean of zero and a standard deviation of one
(5) z-transformed to mean of zero and a standard deviation of one

Table S5. (cont.) Summary of Additional models

	Confidence					Long-Term Effects - Personal					Long-Term Effects - Society				
Predictors	*Estimates*	*std. Error*	*CI*	*t value*	*p*	*Estimates*	*std. Error*	*CI*	*t value*	*p*	*Estimates*	*std. Error*	*CI*	*t value*	*p*
intercept	62.35	0.96	60.46 – 64.07		(1)	57.02	1.07	54.92 – 59.12		(1)	73.96	0.86	72.26 – 75.66		(1)
country (Italy)(2)	-8.80	1.72	-12.24 – -5.30	-5.10	<0.001	2.14	1.91	-1.60 – 5.89	1.12	0.262	5.11	1.54	2.08 – 8.13	3.32	0.001
assessment period (T3)(3)	1.93	1.05	-0.12 – 3.89	1.82	0.069										
age(4)	-0.95	0.91	-2.70 – 0.76	-1.04	0.300	3.75	1.08	1.63 – 5.86	3.48	0.001	0.47	0.87	-1.24 – 2.18	0.54	0.589
fear(5)	-6.06	0.87	-7.83 – -4.37	-6.94	<0.001	5.71	1.06	3.63 – 7.79	5.38	<0.001	4.26	0.86	2.58 – 5.94	4.98	<0.001
country (Italy)*assessment period (T3)	2.02	1.87	-1.63 – 5.63	1.07	0.283										
assessment period (T3)*age	-0.52	0.88	-2.20 – 1.21	-0.59	0.555										
country (Italy)*age	0.82	1.45	-1.97 – 3.49	0.57	0.570	-6.27	1.92	-10.04 – -2.50	-3.27	0.001	-4.32	1.55	-7.36 – -1.29	-2.79	0.005
assessment period (T3)*fear	0.50	0.90	-1.29 – 2.27	0.55	0.580										
country (Italy)*fear	-3.51	1.28	-5.97 – -0.97	-2.73	0.006	0.87	1.89	-2.84 – 4.57	0.46	0.646	-1.21	1.52	-4.20 – 1.78	-0.79	0.428
age*fear	0.49	0.61	-0.68 – 1.79	0.80	0.424	0.83	0.91	-0.95 – 2.62	0.92	0.358	1.14	0.73	-0.30 – 2.57	1.55	0.121

(1) not shown because of being of very limited interpretability
(2) comparison with the reference level (Germany)
(3) comparisons with the reference level (T1)
(4) log- and z-transformed to mean of zero and a standard deviation of one
(5) z-transformed to mean of zero and a standard deviation of one

Table S6. Summary of Reduced Additional models

	Sit. Assessment - Economy					Sit. Assessment - Personal					Behavior Changes					Confidence				
Predictors	*Estimates*	*std. Error*	*CI*	*t value*	*p*	*Estimates*	*std. Error*	*CI*	*t value*	*p*	*Estimates*	*std. Error*	*CI*	*t value*	*p*	*Estimates*	*std. Error*	*CI*	*t value*	*p*
intercept	32.04	0.91	30.22 – 33.87		(1)	47.24	0.80	45.72 – 48.68		(1)	65.32	0.94	63.44 – 67.18		(1)	62.02	0.91	60.26 – 63.82		(1)
country (Italy)[2]	-10.23	1.36	-12.91 – -7.51	-7.48	<0.001	-2.61	1.20	-5.10 – -0.44	-2.17	0.030	4.20	1.66	1.08 – 7.52	2.52	0.012	-7.62	1.42	-10.35 – -4.94	-5.35	<0.001
assessment period (T3)[3]	8.81	0.96	6.85 – 10.58	9.13	<0.001	6.44	0.85	4.64 – 8.12	7.56	<0.001	-13.36	1.02	-15.30 – -11.50	-13.14	<0.001	2.57	0.86	0.81 – 4.16	2.98	0.003
age[4]	0.53	0.80	-1.03 – 2.13	0.67	0.505	-0.51	0.71	-1.89 – 0.77	-0.72	0.471	4.58	0.66	3.33 – 5.96	6.96	<0.001	-1.00	0.67	-2.32 – 0.21	-1.50	0.134
fear[5]	1.54	0.60	0.29 – 2.66	2.57	0.010	-0.59	0.53	-1.61 – 0.38	-1.11	0.265	4.07	0.73	2.71 – 5.65	5.53	<0.001	-5.78	0.73	-7.35 – -4.42	-7.85	<0.001

Note: no reduced model was fitted for Situation Assessment - Health since all predictors were in at least one significant interaction.

(1) not shown because of being of very limited interpretability
(2) comparison with the reference level (Germany)
(3) comparisons with the reference level (T1)
(4) log- and z-transformed to mean of zero and a standard deviation of one
(5) z-transformed to mean of zero and a standard deviation of one

Table S6. (cont.) Summary of Reduced Additional models

	Long-Term Effects - Personal					Long-Term Effects - Society				
Predictors	*Estimates*	*std. Error*	*CI*	*t value*	*p*	*Estimates*	*std. Error*	*CI*	*t value*	*p*
intercept	56.95	1.06	54.86 – 59.04		(1)	73.82	0.86	72.14 – 75.51		(1)
country (Italy)[2]	2.26	1.91	-1.48 – 5.99	1.18	0.237	5.16	1.54	2.14 – 8.18	3.36	0.001
assessment period (T3)[3]										
age[4]	3.64	1.06	1.55 – 5.73	3.42	0.001	0.26	0.86	-1.43 – 1.95	0.30	0.765
fear[5]	6.01	0.87	4.31 – 7.72	6.92	<0.001	3.90	0.70	2.53 – 5.28	5.57	<0.001

(1) not shown because of being of very limited interpretability
(2) comparison with the reference level (Germany)
(3) comparisons with the reference level (T1)
(4) log- and z-transformed to mean of zero and a standard deviation of one
(5) z-transformed to mean of zero and a standard deviation of one
